# Renalase as a Novel Biomarker for Evaluating the Severity of Hepatic Ischemia-Reperfusion Injury

**DOI:** 10.1155/2016/3178562

**Published:** 2016-10-27

**Authors:** Huili Li, Jianrong Guo, Hongli Liu, Yanfeng Niu, Lixia Wang, Kun Huang, Jiliang Wang

**Affiliations:** ^1^Department of Gastrointestinal Surgery, Union Hospital, Tongji Medical College, Huazhong University of Science and Technology, 1277 Jiefang Avenue, Wuhan, Hubei, China; ^2^Cancer Center, Union Hospital, Tongji Medical College, Huazhong University of Science and Technology, 1277 Jiefang Avenue, Wuhan, Hubei, China; ^3^Department of Radiology, Union Hospital, Tongji Medical College, Huazhong University of Science and Technology, 1277 Jiefang Avenue, Wuhan, Hubei, China; ^4^Institution of Cardiology, Union Hospital, Tongji Medical College, Huazhong University of Science and Technology, 1277 Jiefang Avenue, Wuhan, Hubei, China

## Abstract

Hepatic ischemia-reperfusion (I/R) injury is a serious complication in clinical practice. However, no efficient biomarkers are available for the evaluation of the severity of I/R injury. Recently, renalase has been reported to be implicated in the I/R injury of various organs. This protein is secreted into the blood in response to increased oxidative stress. To investigate the responsiveness of renalase to oxidative stress, we examined the changes of renalase in cell and mouse models. We observed a significant increase of renalase expression in HepG2 cells in a time- and dose-dependent manner when treated with H_2_O_2_. Renalase expression also increased significantly in liver tissues that underwent the hepatic I/R process. The increased renalase levels could be efficiently suppressed by antioxidants* in vitro* and* in vivo*. Furthermore, serum renalase levels were significantly increased in the mouse models and also efficiently suppressed by antioxidants treatment. The variation trends are consistent between renalase and liver enzymes in the mouse models. In conclusion, renalase is highly sensitive and responsive to oxidative stress* in vitro* and* in vivo*. Moreover, renalase can be detected in the blood. These properties make renalase a highly promising biomarker for the evaluation of the severity of hepatic I/R injury.

## 1. Introduction

Ischemia is ceasing of blood supply, causing a shortage of oxygen; reperfusion is restoring of blood supply after ischemia. The ischemia-reperfusion (I/R) process occurs in many clinically important events, including hepatic resectional surgery, transplantation, trauma, and hemorrhagic shock [1–3]. Hepatic I/R injury is an inevitable complication causing severe cellular death, tissues damage, and liver dysfunction, which increases the mortality [4–6]. Reactive oxygen species (ROS), which are the main toxicants in oxidative stress, play critical roles in hepatic I/R injury [7–10]. Vascular endothelial cells, hepatic sinusoidal endothelial cells, Kupffer cells, or polymorphonuclear leukocytes are activated by the I/R process and produce a large amount of ROS in oxidative stress process, including superoxide, hydrogen peroxide (H_2_O_2_), hydroxyl radicals, and nitric oxide. This ROS production is the main factor responsible for damaging liver parenchymal cells, increasing vascular permeability, and inducing inflammatory cell infiltration [2, 11–15]. H_2_O_2_ is the most abundant and stable ROS. It leads to oxidative stress and is implicated in a variety of inflammatory diseases [16, 17]. H_2_O_2_ can be converted into hydroxyl radicals, which are extremely reactive and more toxic than other ROS [8, 9]. In this context, H_2_O_2_ is the perfect agent to establish an oxidative stress model in cells and also to simulate I/R injury* in vitro* to some extent.

It has been proposed that I/R injury is an intricate process in which the oxidant/antioxidant balance is changed in favor of the oxidants [18]. Treatment with superoxide dismutase (SOD) and catalase (CAT) reduces the ROS levels, as SOD rapidly dismutases the superoxide (O_2_^−^) to H_2_O_2_, and CAT scavenges H_2_O_2_ to produce water, which reduces the severity of oxidative stress [5]. Beside SOD and CAT, a variety of compounds and biomaterials has been developed as potential therapeutic agents for I/R injury [6, 17]. However, it still lacks efficient, specific, and sensitive biomarkers for the accurate evaluation of the severity of oxidative stress in hepatic I/R injury [19].

Renalase, a ubiquitous flavin adenine dinucleotide-containing amino oxidase, has been implicated in the process of I/R injury [20]. Since first being identified in 2005, renalase has been reported to be synthesized in various organs, including kidney, heart, liver, and adipose tissues [21]. Renalase is secreted into the blood in response to increased oxidative stress [22–24]. The elevated renalase level under stress conditions makes renalase a potential biomarker for the evaluation of the severity of organ I/R injury.

In the present study, we demonstrated that renalase is a sensitive ROS-responsive gene in hepatocytes. In hepatic I/R injury mouse models, renalase was augmented in liver and blood. Moreover, the augmentation of renalase can be ameliorated by antioxidants pretreating, which can reduce the severity of oxidative stress,* in vitro* and* in vivo*. These findings provide evidence that renalase can serve as an efficient and sensitive biomarker for the early warning or evaluation of the severity of hepatic I/R injury.

## 2. Material and Methods

### 2.1. Ethics Statement

All animal experiments were performed in accordance with the National Institutes of Health (NIH) Guide for the Care and Use of Laboratory Animals published by the US National Institutes of Health (NIH Publication, 8th edition, 2011) and approved by the Ethics Committee of Tongji Medical College, Huazhong University of Science and Technology, China.

### 2.2. Reagents and Cell Culture

H_2_O_2_, pentobarbital sodium, SOD, and CAT were purchased from Sigma-Aldrich (St. Louis, MO, USA). Human hepatocellular carcinoma cell line HepG2 was purchased from the American Type Culture Collection (ATCC, Manassas, VA, USA). Cells were maintained in DMEM (high glucose; Invitrogen, Madison, WI, USA) supplemented with 10% FBS (HyClone, Logan, UT, USA) and 100 U/mL penicillin-100 *μ*g/mL streptomycin (Gibco, Carlsbad, CA, USA) and kept in a humidified atmosphere at 37°C with 5% CO_2_ in an incubator (Thermo Fisher Scientific Inc., Waltham, MA, USA).

### 2.3. *In Vivo* Hepatic I/R Model

The* in vivo* hepatic I/R model was performed as previously described [3, 16, 17, 25]. Male C57BL/6 mice, aged 8–12 weeks, were purchased from Beijing University (Beijing, China) and maintained on a chow diet in a 12 h light/12 h dark environment at 25°C in the Animal Care Facility of Tongji Medical College. Surgical procedures on mice were performed under sterile conditions by administration of pentobarbital sodium (50 mg/kg) by an intraperitoneal injection. One hour before the pentobarbital sodium anesthesia, the I/R+SOD+CAT group was intraperitoneally injected with 300 KU/kg SOD and 60 mg/kg CAT, whereas the sham and I/R mice groups were given physiological saline as the solvent by the same method. Laparotomy was performed by vertically opening 2.5–3 cm in the anterior part of the abdomen of the anesthetized mice. After identifying the portal triad and biliary tree, the main trunk of the hepatic artery and portal vein, except for the vasculatures to the right lower lobe, was clamped with a vascular clip to achieve ischemic injury to approximately 70% of the liver. After 1 h of ischemia, reperfusion was achieved by releasing the vascular clip. No vascular clamp was done for the sham group of mice. Then, the incision was closed with silk suture. Six hours after reperfusion, hepatic lobes underwent I/R and the corresponding hepatic lobes from the mice of the sham group were removed and used for further assays. Histological evaluations (H&E staining and IHC of cleaved caspase-3) were performed to quantify the degree of liver injury. Confocal immunofluorescence imaging of frozen sections was performed to detect the renalase levels. Western blotting and real-time qPCR were performed to detect the protein levels and mRNA expression of renalase in liver tissue. Blood was taken by eyeball extirpating and then centrifuged for serum separation, and the serum was used for detection of levels of the renalase and liver enzymes.

### 2.4. Western Blot Analysis

As previously described [26–28], total cells and tissues were lysed using RIPA lysis buffer, and the protein concentration was determined with a BCA protein assay kit (Pierce Company, Rockford, IL, USA). Protein extracts were used for SDS-PAGE (Invitrogen, Carlsbad, CA, USA), and the proteins were transferred to a polyvinylidene fluoride membrane (Millipore, Billerica, MA, USA), which was blocked with 5% nonfat milk in TBS for 3 h and incubated with various primary antibodies overnight at 4°C. After incubation with HRP-conjugated secondary antibodies (diluted 1 : 5000) for 1 h at room temperature, the membranes were treated with ECL reagents (170–5061, Bio-Rad, Hercules, CA, USA) prior to visualization using a ChemiDoc MP imaging analysis system (Bio-Rad, Hercules, CA, USA) according to the manufacturer's instructions. The specific protein expression levels were normalized to *β*-tubulin on the same nitrocellulose membrane. The following primary antibodies and dilutions were used: anti-renalase (GTX89570, diluted 1 : 1000) was purchased from GeneTex (Irvine, CA, USA); anti-*β*-tubulin (sc-9104, diluted 1 : 2000) was purchased from Santa Cruz Biotechnology (Santa Cruz, CA, USA).

### 2.5. Real-Time RT-RCR

As previously described [29, 30], total RNA was isolated using TRIzol reagent (Invitrogen, Carlsbad, CA, USA) according to the manufacturer's instructions. 2 *μ*g of total RNA was reversely transcripted using an RNA PCR Kit (Takara Biotechnology, Otsu, Japan), and the resulting cDNA was used as a PCR template. The mRNA levels were determined by real-time qPCR with an ABI PRISM 7900 Sequence Detection System (Applied Biosystems, Foster City, CA, USA) according to the manufacturer's instructions, and *β*-tubulin was used as endogenous control. The experiment was performed in triplicate. The relative gene expression levels were calculated using the comparative C_T_ method applying the formula 2^−ΔΔC_T_^. The primer sequences for real-time qPCR are listed in Table 1.

### 2.6. Confocal Immunofluorescence

Fresh ischemia-reperfusion hepatic lobes were collected and rinsed in saline to remove remaining blood. Tissues were cut into 6-*μ*m thick sections with a freezing slicing microtome. The sections were then immersed and fixed in 4% paraformaldehyde at room temperature for one-half hour. Afterwards, the sections were incubated with 5% bovine serum albumin (BSA) and immunolabeled with the indicated antibody (renalase GTX89570, GeneTex) at 4°C overnight. After washing, the sections were incubated with Alexa Fluor 594 goat anti-rabbit IgG (R37117, Invitrogen) for 1 h. After washing, 4′,6-diamidino-2-phenylindole (DAPI) was added to stain the cell nuclei on ice. Tissue fluorescence was imaged on a confocal microscope (Alsi, Nikon). For the quantitative expression of renalase, the density of fluorescence was analyzed by the ImageJ 1.44p software.

### 2.7. Liver Histology and IHC

Formalin-fixed liver specimens were embedded in paraffin blocks and cut into 5 *μ*m sections. The sections were then stained with hematoxylin and eosin for histology. For IHC, the sections were deparaffinized and rehydrated with ethanol and xylene and were heated to 95–98°C for 20 min in 10 mM citrate buffer, pH 6.0. After blocking with PBS containing 10% goat serum for 1 h at room temperature, the sections were incubated with a primary antibody 1 : 200 cleaved caspase-3 overnight at 4°C. After incubation with secondary antibodies, the sections were mounted and evaluated with an Olympus microscope.

### 2.8. Detection of Liver Enzymes in Serum

Alanine transaminase (ALT), aspartate transaminase (AST), gamma-glutamyl transpeptidase (GGT), alkaline phosphatase (ALP), and lactate dehydrogenase (LDH) in serum were determined using automatic biochemical analyzer (TBA-40FR, Toshiba, Tokyo, Japan). Related detection kits were purchased from MedicalSystem Biotechnology Co., Ningbo, China.

### 2.9. Detection of Serum Renalase Protein Levels

The protein levels of renalase in the serum were determined by using a Mouse Renalase (RNLS) ELISA Kit (MU30925, Bio-Swamp, Wuhan, China) according to the manufacturer's instructions. Briefly, dilute standard first. Add serum 40 *μ*L to testing well, then add biotinylated anti-RNLS-antibody 10 *μ*L, and gently mix. Incubate for 30 min at 37°C. Dry and wash every well. Add HRP-Conjugate Reagent 50 *μ*L to each well, except the blank well. Incubate and wash. Then add chromogen solutions and incubate for 15 min at 37°C. Measure the optical density (OD) at 450 nm after adding Stop Solution within 15 min. Calculate according to the manufacturer's instructions.

### 2.10. Statistical Analysis

As described in previous studies [31, 32], the statistical analysis was performed with the Statistical Package for Social Sciences (SPSS version 13.0; IBM Analytics, Chicago, IL, USA). All the data were expressed as mean ± SD (standard deviation, SD) and the difference was analyzed by a one-way ANOVA test. Statistical analysis was performed using Student's* t*-test for paired data. The difference was considered statistically significant for *p* < 0.05.

## 3. Results

### 3.1. Increase of the Renalase Expression in HepG2 Cells Induced by H_2_O_2_

**Figure 1:**
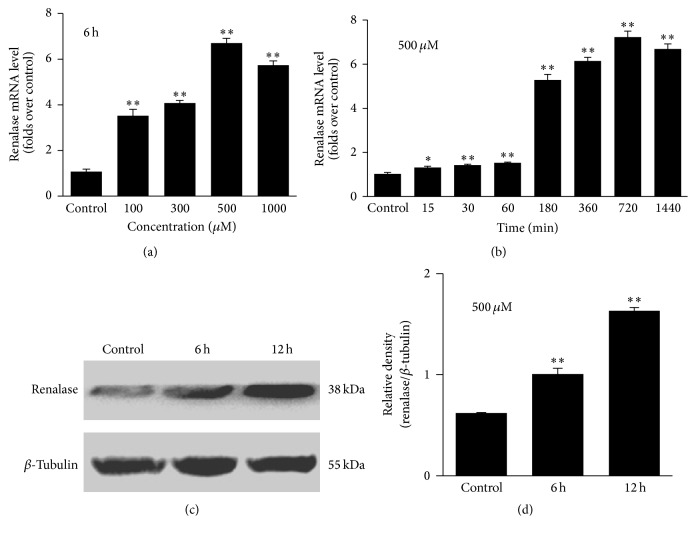
Increase of renalase expression in hepatic cells upon H_2_O_2_ treatment. (a) Relative expression of renalase mRNA evaluated by real-time qPCR in HepG2 cells treated with increasing concentrations of H_2_O_2_. (b) Relative expression of renalase mRNA evaluated by real-time qPCR in HepG2 cells treated with H_2_O_2_ for different times. (c) Western blot analysis of renalase protein levels in HepG2 cells treated with 500 *μ*M H_2_O_2_ for 6 h or 12 h. (d) Densitometric analysis of Western blot of renalase in HepG2 cells treated with 500 *μ*M H_2_O_2_ for 6 h or 12 h. ^*∗*^ *p* < 0.05; ^*∗∗*^ *p* < 0.01, compared with control group. Data are plotted as the mean ± SD from five independent experiments. Bars indicate the standard deviation of the mean.

To investigate the impact of oxidative stress on the renalase expression in HepG2 cells, the cells were incubated with H_2_O_2_ in different concentrations and for different times. First, HepG2 cells were incubated with H_2_O_2_ in increasing concentrations (0 *μ*M, 100 *μ*M, 200 *μ*M, 500 *μ*M, and 1000 *μ*M) for 6 h, respectively. With increasing H_2_O_2_ concentration, the mRNA expression of renalase increased proportionally (Figure 1(a)). The maximal expression of renalase was detected for a concentration of 500 *μ*M. Renalase expression decreased slightly when incubated with a concentration of 1000 *μ*M compared with 500 *μ*M (Figure 1(a)). This suggests that the concentration of 1000 *μ*M may be beyond the cell's affordable range and causes cell death. We used relatively higher concentrations (1000 *μ*M, 2000 *μ*M, 3000 *μ*M, and 5000 *μ*M) of H_2_O_2_ to treat cells and found that cell death increased with the increasing concentrations of H_2_O_2_. Treating with 2000 *μ*M of H_2_O_2_ for 6 h could even cause more than half of the cell death. No cells could survive in the concentration of 5000 *μ*M H_2_O_2_ (data not shown). Then, HepG2 cells were incubated with 500 *μ*M H_2_O_2_ for different times (15 min, 30 min, 1 h, 3 h, 6 h, 12 h, and 24 h). Increasing the incubation time induced a proportional increase of the mRNA expression of renalase (Figure 1(b)). The maximal expression of renalase was measured for an incubation time of 12 h. Renalase expression decreased slightly when incubated for 24 h (Figure 1(b)). This indicates that the cells may not tolerate the long-time incubation of H_2_O_2_. Further, the protein levels of renalase in HepG2 cells that were incubated with 500 *μ*M H_2_O_2_ for either 6 h or 12 h were measured and found to be larger for the longer incubation (*p* < 0.01, Figures 1(c) and 1(d)).

### 3.2. Suppression of H_2_O_2_-Induced Augmentation of the Renalase Expression in HepG2 Cells Mediated by Antioxidant Preincubation

**Figure 2:**
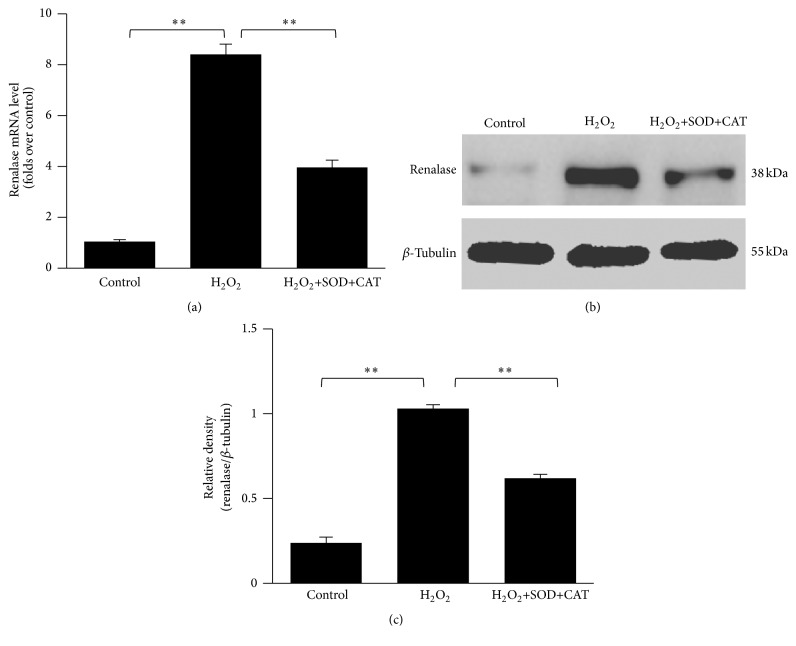
Antioxidant-induced decrease of H_2_O_2_-induced augmentation of the renalase expression in hepatic cells. (a) Relative expression of renalase mRNA evaluated by real-time qPCR in H_2_O_2_-incubated (12 h) HepG2 cells with or without pretreatment with antioxidants. (b) Western blot analysis of renalase protein levels in H_2_O_2_-incubated (12 h) HepG2 cells with or without preincubation with antioxidants. (c) Densitometric analysis of Western blot of renalase in H_2_O_2_-incubated HepG2 cells with or without preincubation with antioxidants. ^*∗∗*^ *p* < 0.01. Data are plotted as the mean ± SD from five independent experiments. Bars indicate the standard deviation of the mean.

Antioxidants can reduce the severity of oxidative stress. To investigate whether the H_2_O_2_-induced augmentation of the renalase expression in HepG2 cells can be decreased by antioxidants, cells were preincubated with 300 KU/L SOD and 60 mg/L CAT for 2 h. Before the H_2_O_2_-treatment, the SOD and CAT-containing medium was replaced by FBS-free medium. The obtained results showed that the SOD/CAT preincubation significantly decreased the H_2_O_2_-induced augmentation of the renalase mRNA expression in HepG2 cells (*p* < 0.01, Figure 2(a)). Furthermore, the SOD/CAT preincubation significantly decreased the H_2_O_2_-induced increase of the renalase protein levels in HepG2 cells (*p* < 0.01, Figures 2(b) and 2(c)).

### 3.3. Increased Renalase Expression and Histologic Damage in Hepatic I/R Injury Mouse Models and the Suppression to the Increased Renalase Expression by Antioxidants

**Figure 3:**
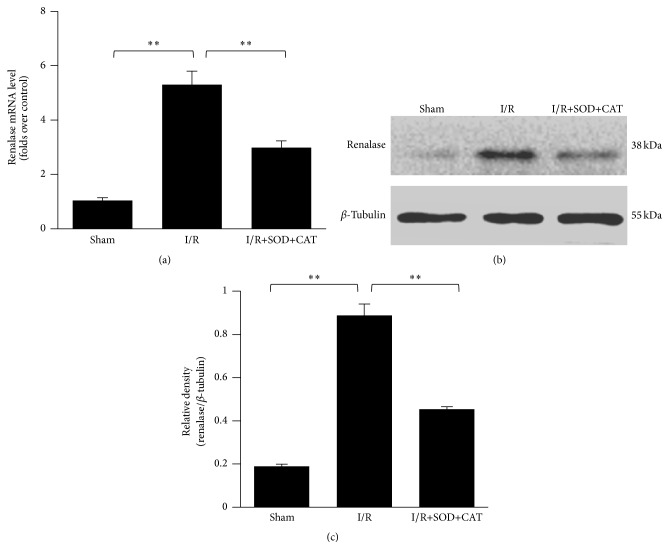
Augmentation of renalase expression in hepatic I/R injury models and the subsequent decrease induced by antioxidant preinjection. (a) Relative expression of renalase mRNA evaluated by real-time qPCR in livers of mouse models (sham, I/R injury, and I/R injury with antioxidant preinjection, 6 h after reperfusion). (b) Western blot analysis of renalase protein levels in livers of mouse models (sham, I/R injury, and I/R injury with antioxidant preinjection, 6 h after reperfusion). (c) Densitometric analysis of Western blot of renalase in livers of mouse models (sham, I/R injury, and I/R injury with antioxidant preinjection, 6 h after reperfusion). ^*∗∗*^ *p* < 0.01. Data are plotted as the mean ± SD from five independent experiments. Bars indicate the standard deviation of the mean.

**Figure 4:**
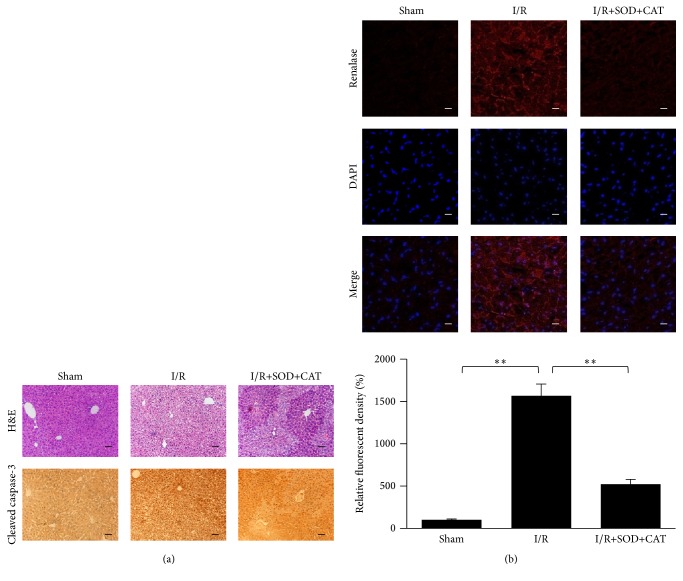
Increase of renalase levels in hepatic I/R injury models and its suppression induced by antioxidant preinjection and the corresponding histological changes in liver. (a) The livers of the sham, I/R, and IR+SOD+CAT groups (6 h after reperfusion) were subjected to histological evaluation by H&E staining and IHC of cleaved caspase-3. Scale bar (black) represents 50 *μ*m. (b) Confocal immunofluorescence imaging of renalase in livers of mouse models (sham, I/R injury, and I/R injury with antioxidant preinjection, 6 h after reperfusion). Scale bar (white) represents 30 *μ*m. The relative fluorescent density of each group was analyzed. ^*∗∗*^ *p* < 0.01. Data are plotted as the mean ± SD from five independent experiments. Bars indicate the standard deviation of the mean.

In hepatic I/R injury mouse models, the renalase mRNA expression in livers was significantly augmented (*p* < 0.01, Figure 3(a)), and the intraperitoneal preinjection of SOD and CAT decreased this augmentation significantly (*p* < 0.01, Figure 3(a)). The renalase protein levels in livers of the mouse models were significantly increased (*p* < 0.01, Figure 3(c)) and could also be suppressed by the intraperitoneal preinjection of SOD and CAT (*p* < 0.01, Figure 3(c)). This result was confirmed by Western blot (Figures 3(b) and 3(c)) and confocal immunofluorescence imaging (Figure 4(b)). The histological evaluation revealed that I/R caused hydropic degeneration and necrosis of hepatic tissue and cells. A significant improvement was observed in I/R+SOD+CAT group (Figure 4(a)). Consistent with the observed hydropic degeneration and necrosis during I/R, hepatic apoptosis was increased as seen by the brown intracellular staining of cleaved caspase-3 in most hepatocytes. A significant reduction of cleaved caspase-3 staining was observed in the I/R+SOD+CAT group (Figure 4(a)).

### 3.4. Increase of the Serum Levels of Renalase and Liver Enzymes in Hepatic I/R Injury Mouse Models and the Suppression to the Increased Renalase and Liver Enzymes by Antioxidants

**Figure 5:**
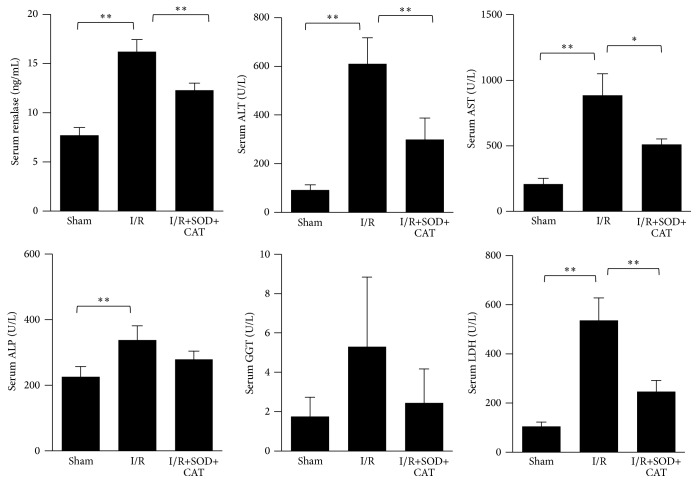
Increase of serum renalase and liver enzymes levels in hepatic I/R injury models and its suppression induced by antioxidant preinjection. Serum renalase and liver enzymes: ALT, AST, GGT, ALP, and LDH levels in mouse models (sham, I/R injury, and I/R injury with antioxidant preinjection, 6 h after reperfusion). ^*∗*^ *p* < 0.05; ^*∗∗*^ *p* < 0.01. Data are plotted as the mean ± SD from five independent experiments. Bars indicate the standard deviation of the mean.

The liver enzymes (ALT, AST, GGT, ALP, and LDH) and renalase levels were measured in the serum of the hepatic I/R injury mouse models. The renalase levels were found to be significantly augmented in the hepatic I/R process (*p* < 0.01). The levels of ALT (*p* < 0.01), AST (*p* < 0.01), ALP (*p* < 0.05), and LDH (*p* < 0.01) elevated significantly in the hepatic I/R group compared to the sham group, whereas GGT increased slightly in the hepatic I/R group and with no statistical significance to the sham group (*p* > 0.05). The increase of ALT, AST, LDH, and renalase levels could be effectively suppressed (renalase, ALT, and LDH: *p* < 0.01; AST: *p* < 0.05) by the intraperitoneal preinjection of SOD and CAT. ALP and GGT could also be suppressed by antioxidants, but with no statistical significance (*p* > 0.05) to the I/R group (Figure 5).

## 4. Discussion

Hepatic I/R injury may occur in hepatic resections, liver transplantations, traumas, and vascular surgeries [33–35]. It is a serious complication in clinical practice. The redox balance in the process of hepatic I/R injury leads to accumulation of ROS, which presents the disease mechanism most commonly invoked in hepatic I/R injury [33]. Suppressing ROS-burst in oxidative stress is an efficient way to alleviate hepatic I/R injury. However, early warning, evaluation of the severity, or therapeutic effect of oxidative stress, which is even more important in clinical practice of hepatic I/R injury management, is a remaining challenge. This is due to the lack of efficient, specific, and sensitive biomarkers for the accurate evaluation of the severity of oxidative stress [19]. Biochemical markers used to evaluate the severity of oxidative stress include malondialdehyde (MDA) [36, 37], ascorbic acid (AA)/dehydroascorbic acid (DHA) [38, 39], and a series of inflammatory, proinflammatory, or anti-inflammatory biomarkers [40–42]. However, these markers face various limitations. MDA is a marker for lipid peroxidation, which usually proceeds in the I/R injury of the extremities [43]. A close relationship between MDA and cardiac necrosis markers has been reported [36], but whether the MDA level can reflect the severity of oxidative stress or I/R injury of other organs remains unclear. The determination of AA and DHA levels is challenging because of the unstable nature of these compounds [39]. Inflammatory, proinflammatory, and anti-inflammatory markers cover a wide range of cytokines. However, these cytokines are not specific for evaluating oxidative stress. Therefore, an efficient, specific, and sensitive biomarker for evaluating oxidative stress is still urgently needed.

The human renalase gene is located on chromosome 10 at q23.33 and encodes the conserved protein renalase consisting of 342 amino acids. Renalase was reported to efficiently degrade dopamine and epinephrine in the blood [21], and it has been verified that it is the only enzyme involved in the catecholamine metabolism that can be secreted into the blood cycle hitherto [44]. Previous studies indicated that renalase is multifunctional and closely related to oxidative stress conditions such as stroke, heart transplantation, or acute kidney injury [45–48]. Considering its tight relationship with oxidative stress, renalase is thought to play a role, or at least responsive, in the process of hepatic I/R injury.

In particular, H_2_O_2_ is considered as the most abundant and stable ROS. H_2_O_2_ is a mild oxidant but is converted into hydroxyl radicals, which are extremely reactive and more toxic than other ROS [49, 50]. Therefore, to investigate the responsiveness of renalase in hepatic cells under the condition of oxidative stress, H_2_O_2_ was used to mimic the ROS-burst in oxidative stress. For the cell model* in vitro*, the common hepatocellular carcinoma cell line HepG2 was chosen. Renalase expression in HepG2 cells showed a positive correlation with H_2_O_2_ concentration and incubation time. These results suggest that renalase is responsive to the ROS-burst or oxidative stress in hepatic cells, and it is sensitive to the degree or severity and duration of oxidative stress.

Disturbance of the redox balance is characteristic for oxidative stress in the process of the hepatic I/R injury, which leads to ROS-burst and antioxidant consumption [33]. The most efficient way to ameliorate oxidative stress is replenishing ROS-scavenging antioxidants [17]. SOD is a common antioxidant, which is wildly distributed within mammalian organisms [51]. It is an important free radical scavenger, which has been clinically used in several countries. CAT is also an endogenous antioxidant, which can catalytically decompose H_2_O_2_ [52, 53], but has not been used in clinical practice. In this study, SOD and CAT were used for the preincubation of the cells. We observed that the SOD/CAT preincubation significantly ameliorated the elevation of the renalase expression in HepG2 cells. The application of antioxidants reduced the severity of oxidative stress, and the responsive elevation of the renalase expression was ameliorated accordingly. This result suggests that renalase is responsive to the therapeutic means for oxidative stress in hepatic cells. And the expression of renalase is closely related to the degree or severity of the oxidative stress.

The hepatic I/R injury mouse model is the most commonly used animal model in various studies for hepatic I/R injury and oxidative stress* in vivo*. We used this model to investigate whether renalase is responsive to the hepatic I/R injury* in vivo*. Renalase expression was significantly increased in the livers of hepatic I/R injury mouse models. Intraperitoneal preinjection of SOD and CAT ameliorated the elevation of renalase in the livers significantly. Furthermore, a consistent high-and-low variation between the renalase expression in liver and the degree of liver damage, including tissue necrosis and cell apoptosis, has been observed. Liver enzymes are the most important evaluation indexes in the standard liver function test (LFT) panel. Serum levels of liver enzymes, including ALT, AST, GGT, ALP, and LDH, were increased by different degrees in the hepatic I/R injury mouse models. Consistently, the serum renalase levels of the hepatic I/R injury mouse models were significantly elevated. The elevations of serum levels of either the renalase or the liver enzymes could be significantly reduced by the intraperitoneal preinjection of SOD and CAT. These results suggest that renalase is responsive to hepatic I/R injury and therapeutic means. Its expression in liver tissue closely related to the severity of oxidative stress and the subsequent liver damage. Moreover, the serum renalase level can sensitively reflect the severity and therapeutic effect of oxidative stress in the process of hepatic I/R injury. Combining with the standard LFT panel, more accurate and specific evaluations of the hepatic I/R injury can be made.

In conclusion, to the best of our knowledge, this study firstly demonstrates the value and applicability of renalase as a promising biological marker for the evaluation of the severity of oxidative stress in hepatic I/R injury. We investigated the responsiveness of renalase to conditions related to oxidative stress* in vitro* and* in vivo*. Our results revealed that oxidative stress efficiently induces the elevation of renalase expression* in vitro* and* in vivo*. Furthermore, this elevated renalase expression can be decreased by antioxidants. Its sensitive responsiveness to the severity of oxidative stress and convenient detection in the blood make renalase an ideal biomarker for the evaluation of the severity and therapeutic effect of hepatic I/R injury. In the future, clinical treatment decisions based on the detection of renalase activities in the blood may help to improve the clinical outcomes of hepatic resections, liver transplantations, traumas, or vascular surgeries. Nevertheless, the variation trend of serum renalase level in the hepatic I/R process under different conditions and time points has not been demonstrated in this study, which needs further investigations. Beside the I/R injury, there are various pathophysiological processes which are related to oxidative stress. Renalase may play roles in these processes, and the variation trend of renalase expression in these processes may be different from each other. Related researches may make renalase serve as a novel inspection item, which can assist the existing inspection items to make the evaluations of the diseases more accurate. Other potential applications of renalase in clinical practice require further explorations and investigations.

## Figures and Tables

**Table 1:** The sequences of primers for real-time qPCR.

Name	Use	Orientation	Sequence
Renalase	Real-time qPCR	F	5′-AGTGAACGCCAGAGGGAGCAA-3′
		R	5′-TAGCGGCAGGACCAAGGGAC-3′
*β*-Tubulin	Real-time qPCR	F	5′-GCTCATCGCTTATCACCTCC-3′
		R	5′-GAGCGCTCTGTCCACGTACT-3′
